# A scoping review of non-communicable disease research capacity strengthening initiatives in low and middle-income countries

**DOI:** 10.1186/s41256-019-0123-1

**Published:** 2019-11-29

**Authors:** Tilahun Nigatu Haregu, Allison Byrnes, Kavita Singh, Thirunavukkarasu Sathish, Naanki Pasricha, Kremlin Wickramasinghe, Kavumpurathu Raman Thankappan, Brian Oldenburg

**Affiliations:** 1 Melbourne School of Population and Global Health & WHO Collaborating Centre on Implementation Research for Prevention & Control of NCDs, University of Melbourne, Melbourne, Australia; 2 Family Life Limited, Sandringham, Australia; 3 Centre for Chronic Disease Control, New Delhi, India; 4 Population Health Research Institute, McMaster University, Hamilton, Canada; 5 Burnet Institute, Melbourne, Australia; 6 World Health Organization, Moscow, Russia; 7 Sree Chitra Tirunal Institute for Medical Sciences and Technology, Trivandrum, India

**Keywords:** Non-communicable diseases, Research capacity strengthening, Low and middle-income countries

## Abstract

**Introduction:**

As the epidemic of non-communicable diseases (NCDs) is rapidly developing in low and middle-income countries (LMICs), the importance of local research capacity and the role of contextually relevant research in informing policy and practice is of paramount importance. In this regard, initiatives in research capacity strengthening (RCS) are very important. The aim of this study was to review and summarize NCD research capacity strengthening strategies that have been undertaken in LMICs.

**Methods:**

Using both systematic and other literature search, we identified and reviewed NCD-RCS initiatives that have been implemented in LMICs and reported since 2000. Information was extracted from published papers and websites related to these initiatives using a semi-structured checklist. We extracted information on program design, stakeholders involved, and countries of focus, program duration, targeted researchers, disease focus, skill/capacity areas involved and sources of funding. The extracted information was refined through further review and then underwent a textual narrative synthesis.

**Results:**

We identified a number of different strategies used by research capacity strengthening programs and in the majority of initiatives, a combination of approaches was utilized. Capacity strengthening and training approaches were variously adapted locally and tailored to fit with the identified needs of the targeted researchers and health professionals. Most initiatives focused on individual level capacity and not system level capacity, although some undoubtedly benefited the research and health systems of LMICs. For most initiatives, mid-term and long-term outcomes were not evaluated. Though these initiatives might have enhanced research capacity in the immediate term, the sustainability of the results in the long-term remains unknown.

**Conclusion:**

Most of NCD-RCS initiatives in LMICs focused on building individual capacity and only a few focused explicitly on institutional level capacity strengthening. Though many of the initiatives appear to have had promising short-term outcomes, evidence on their long-term impact and sustainability is lacking.

## Introduction

The World Health Organization’s (WHO) P*rioritized Research Agenda for the Prevention and Control of Non-communicable Diseases (NCDs)* identified key areas of research relating to the prevention and control of NCDs [1]. The United Nations Political Declaration on Prevention and Control of NCDs, which has research and development among the major focus areas, recognized the presence of cost-effective interventions for NCD prevention and the numerous opportunities for global action [2]. The Global Action Plan for the prevention and control of NCDs 2013–2020 included the promotion and support for national capacity for high-quality research for the prevention and control of NCDs as one of its objectives [3]. This action plan called upon international partners to take coordinated action to attain nine voluntary global NCD targets by 2025. The recent Sustainable Development Goals (SDGs) also reaffirm the need for research on NCDs that primarily affect low and middle-income countries (LMICS) [4].

In order to achieve these goals, strengthening research capacity in developing countries is critical. Research capacity strengthening (RCS) is a complex concept that can be understood and interpreted in many ways. According to ESSENCE on Health Research, an initiative of funding agencies to improve the coordination and harmonization of research capacity investments, RCS refers to *“any efforts to increase the ability of individuals and institutions to undertake high-quality research and to engage with the wider community of stakeholders* [5]*.”* Often, RCS is considered as a continuous process both at individual and institutional level and it has been difficult to ascertain the outcomes and define attribution and contribution. It is presumed that successful RCS interventions need to consider individual, institutional and systems components of capacity strengthening [5–9].

The epidemic of NCDs is rapidly emerging in many LMICs. Current evidence shows that 80% of NCD -related deaths occur in LMICs and, more than three quarters of ‘premature’ NCD deaths occur in LMICs [10]. As the magnitude of the epidemic in these countries is expected to increase in the coming years, increasing evidence is being generated on the nature and the scale of this epidemic, the characteristics of the various risk factors, and the social and economic impacts of NCDs [11–13].

However, in many LMICs, the national and local level responses to NCDs are based on evidence generated from elsewhere or adapted from that of communicable diseases. Most of the global policy recommendations are based on studies from high income countries or even when data comes from LMICs, studies are led by researchers in high-income countries [14]. Therefore, the role of locally relevant NCD research to inform policy and practice will be of paramount importance in LMICs [1]. In this regard, strengthening the research capacity of local researchers who are best suited to generate locally relevant evidence is critical. However, there is more rhetoric than reality on capacity strengthening of early and mid-career researchers in LMICs [15, 16].

Prior to recent years, the typical model involved research trainees undertaking the majority of their research training in high income countries and that many of these individuals did not return to their own countries on a full-time basis. Some studies have shown that more than half of those trained in developed countries didn’t return to their country though some would still be conducting research in their own country despite living abroad [17, 18]. Accordingly, overseas scholarships/fellowships that encourage trainees to return home or establish collaboration with home institutions are being considered [19].

A limited number of NCD research capacity strengthening initiatives has been implemented in LMICs. Anecdotal evidence suggests that these initiatives are often externally supported and short-lived [20–22]. On the other hand, some argue that LMICs do not need stand-alone RCS programs dedicated to NCDs as any RCS on health can also address NCDs. However, RCS initiatives specific to NCDs would be more beneficial to the prevention and control of NCDs than the generic RCS focusing on public health. This is due to the fact that the nature of the NCDs, their responses and their causes and consequences depends on local contexts and there is a strong need to have local capacity to generate contextually relevant research to reduce the evidence-implementation gap [23]. Most of the cost-effective interventions for NCDs need involvement of non-health sectors, are complex in nature and need to follow a life course approach [24, 25]. This assertion is taking into consideration the need for appropriate level of integration, where possible.

To realize global, regional and country level goals in the prevention and control of NCDs, LMICs need better research-based evidence than is currently available. For instance, while implementing and evaluating WHO ‘Best Buys’ is needed most LMICs have not conducted research on these interventions in their populations [26]. To generate and apply such evidence in policy and practice strong research capacity is critical. Therefore, the aim of this review was to review and summarize existing capacity strengthening strategies used by NCD research capacity strengthening initiatives. Additionally, this review investigated the approaches and implementation strategies aimed to improve NCD research capacity in LMICs.

## Methods

### Study design

We conducted a systematic review of specific NCD-RCS initiatives that have been implemented in LMICs. This review focused on RCS initiatives that deliberately and systematically addressed NCD research capacity in LMICs. We customized the PRISMA guideline in conducting the review and reporting the findings as the research theme is not a best fit for a typical systematic review.

### Study settings

This study has focused on NCD-RCS initiatives in LMICs although the majority of initiatives have been undertaken in collaboration with universities and research institutions from high income countries. The World Bank’s income -based classification (2017) was used for defining LMICs with most being in Africa, Asia, Latin America and Eastern Europe [27] .

### Study period

Evidence about NCD-RCS initiatives published between January 1, 2000 and July 31, 2017 were considered in this study. This time horizon was selected because most RCS initiatives commenced after 2000 along following the launching of Global strategy for the prevention and control of noncommunicable diseases [28]. The study included both NCD (generic), disease-specific, and risk-factor specific RCS initiatives.

### Search strategy

A systematic literature search with key words (“Non-communicable disease,” “Research Capacity Strengthening/building,” and “Low and Middle-income countries”) combined with a retrospective search from references of papers reporting identified initiatives and a snowballing technique was applied (Table 4 in Appendix 1). We searched Embase, Pubmed and Google scholar for relevant articles. In addition, after identifying an initiative from these and a general search engine, we collected all relevant information relating to that initiative from all possible sources including the initiative’s website, media releases, websites of the funding agencies, and other collaborating institutions for the projects they had funded/implemented. A few NCD researchers from LMIC institutions were also contacted to suggest any remaining initiatives and/or also verify the information about the included initiatives.

### Inclusion/exclusion criteria

Four major inclusion criteria were used to select NCD-RCS initiatives in a progressive manner. These were:

1. Focused primarily on NCDs and/or associated NCD risk factors;
2. Implemented or being implemented in LMICs;
3. Implementation period was between Jan 2000 and July 2017, including ongoing initiatives; and
4. Initiative lasting for a duration of at least 1 year. Initiatives meeting the inclusion criteria and having basic information to describe the model used by the initiative were included.

For bigger NCD-RCS initiatives which had multiple projects, examples of projects that best illustrate the core RCS approach were included.

### Extraction of data

A semi-structured check-list (Table 5 in Appendix 3), with the main headings presented in Table 1, was used to extract and collate information obtained from the available sources. The key variables extracted from the information sources include title of the initiative, implementing and collaborating institutions, year of implementation, the disease/risk factor addressed, the targeted researchers/trainees, focus countries, capacity/skills areas addressed, the model/approach/system put in place to implement the initiative and the funding body. While only key information was extracted for most of these variables, more detailed information was extracted for approaches of RCS. TH and AB did the review of initiatives and extraction of information.

**Table 1:** Characteristics of NCD-RCS initiatives included in this study

SN	NCD-RCS Initiatives	Implementers	Region and countries	Period	Target researchers	Disease focus	Skill areas	Funding support
1	TOBAC Program [29]	29 individual TOBAC projects by various implementers	All regions of LMICs	2002–2012	Early and mid-career researchers	Tobacco use	Research skills	NIDA, NHLBI, FIC
	1.1 Building GIS Capacity in Tobacco research	Loma Linda University	Cambodia, Lao, Mongolia	2002–2010	Tobacco control workforce	Tobacco use	GIS skills in research	NIH/FIC
	1.2 Analytical capacity building for study of Tobacco carcinogen	University of Minnesota; Tata Memorial Center; Healis-Sekhsaria Institute for Public Health	India	2017–2022	Early and mid-career researchers	Tobacco exposure	Analytical capacity	NIH/FIC
	1.3 Tobacco Cessation training and Research in India and Indonesia	University of Minnesota, University of Arizona, Sree Chitra Tirunal Institute for Medical Sciences and Technology, Trivandrum, India and Gaja Mada University Indonesia	India, Indonesia	2002–2007	Tobacco cessation researchers	Tobacco cessation	Tobacco Research	NIH/FC
	1.4 Building Capacity for Tobacco Cessation in India and Indonesia	University of Arizona, Sree Chitra Tirunal Institute for Medical Sciences and Technology, Trivandrum, India and Gaja Mada University Indonesia	India, Indonesia	2008–2013	Tobacco cessation researchers	Tobacco cessation	Building research capacity	NIH/FC
2	GACD research Network [30]	Global Alliance for Chronic Diseases and member institutions	Global but focus on LMICs	2007-	Researchers in LMICs	Chronic disease	Individual and institutional level	Various
3	3.1. CNCD-Africa [31]	CNCD Consortium	Africa	2009	Members of consortium	NCDs	NCD prevention and control	CDC, IUPHE
	3.2. Training Health Researchers into Vocational Excellence (THRiVE) [32]	THRiVE Consortium	African and UK universities	2009	Members of consortium	Generic	Academic rsearchers	Wellcome Trust
4	YP-CDN: NextGen leaders [33]	YP-CDN: Self-initiated network of professionals	International	2009-	Young professionals	Chronic diseases	Individual and network level	RTI
5	Training and Capacity Building in LMIC for Research in Heart and Lung Disease [34]	NHLBI-UnitedHealth Collaborating Centres of Excellence (COE)	International – 10 countries	2009–2014	Early stage investigators (ESIs)	Heart and lung disease	Clinical and public health-related research	NHLBI, NIH, HHS
6	NCD-Lifespan [35]	US institutions and institutions in LMICs	All regions of LMICs	2010 -	Early and mid-career researchers	NCDs	Individual and institutional level	FIC/NIH
	6.1: ASCEND [36]	Monash University, University of Melbourne	South East Asia	2010–2015	Early career researchers with at least Masters (or equivalent experience)	NCDs	NCD research & networking	FIC/NIH
	6.2: Wits RLTP	Wits University	Africa Region	2010–2015	Masters, PhD, postdocs	NCDs	NCD research	FIC/NIH
	6.3 Strengthening Nurse NCD Research and Training Capacity in Thailand	University of Michigan; Benjaporn Rajataramya; Praboromarachanok Institute	Thailand	2014–2019	Pre and post-doctoral	NCDs	NCD research	FIC/NIH
7	APCDR [37]	APCDR along with 18 partner centres including in US and UK	10 countries in Africa	2010 -	Early and mid-career researchers	NCDs	Individual, institutional	MRC
8	Instituto de Cancerología (INCAN) [38]	INCAN.; School of Medicine of Washington University in Saint Louis; Cancer Control Research Training Institute	Guatemala	2010–2012	Clinician researchers	Cancer	Individual and institutional	FIC/NIH
9	AWI-gen [39]	Wits University, INDEPTH network	SSA countries	2012–2017	Early and mid-career researchers	Cardio-metabolic	Individual and institutional	NIH
10	ANPPA [40]	African Population and Health Research Centre; partner institutions	Five countries in Africa	2013–2017	Mid-career researchers	NCDs	Policy analysis	IDRC
11	KSN-NCD [41]	MOH (Kenya), APHRC and other partners	Kenya	2014-	Policy makers, health planners, researchers,	NCDs	Multiple levels	IDRC
12	AACR-AORTC [42]	African Organization for Research and Training in cancer; American Association for Cancer Research	Africa	2015 - present	Health professionals, advocates, and leaders	Cancer	Cancer research	AACR
13	ENCORE Program [43]	University of Melbourne, Australia; PHFI, CCCC, AIIMS, SCTIMST, India	Australia and India	2015–2018	Early and mid-career researchers	NCDs	Research skills	The University of Melbourne
14	Africa wide NCD Research Group [44]	The East African NCD Alliance	Africa	Jan 2017	Experts in different NCD specialties	NCDs	Network capacity	Various – including IDRC

Abbreviations: see list of abbreviation in supplemental material

### Analysis and synthesis of information

A three-stage textual narrative synthesis approach were used [45]. In the first stage, a *horizontal* description and analysis of each NCD-RCS initiative was conducted. At this stage, we summarized information for each initiative. In the second stage, a *vertical* analysis and synthesis of information for each parameter and across the initiatives was conducted. We summarized and presented information for each parameter at this stage. In the final stage, a *diagonal* approach looking across initiatives and parameters as well as how the findings fit with the main principles of RCS was performed. Findings were presented using tables and narrative summaries. An interpretive synthesis approach was used to identify and describe the models.

## Results

### Description of NCD-RCS initiatives

**Fig. 1:**
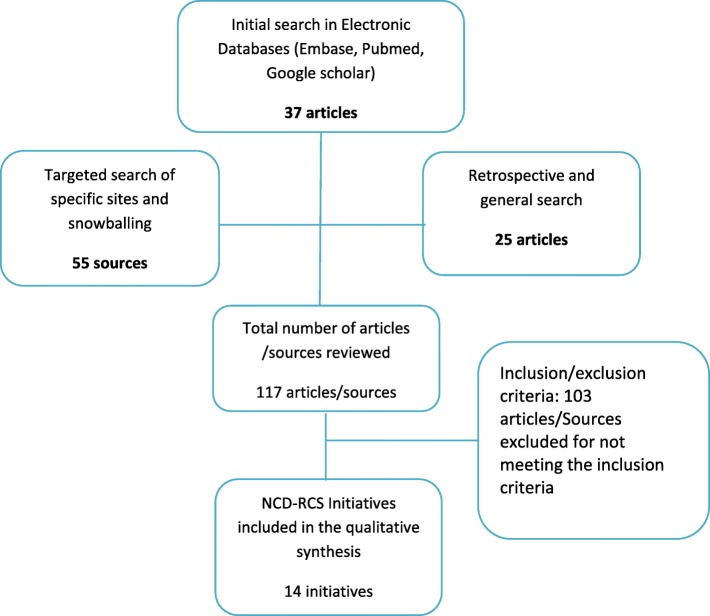
Flow chart describing the selection of initiatives

A total of 14 NCD-RCS initiatives in LMICs that met our inclusion criteria were formally reviewed in this study (see Fig. 1 in Appendix 2). Table 1 presents a detailed description of these initiatives. In brief, most of these initiatives were arranged as a collaboration between developed countries (as sources of funding and as a prime implementing partner) and institutions based in LMICs (as sub-grantee or local partner). US (National Institutes of Health/Fogarty International Centre), Canada (International Research Development Centre) and Australia (National Health and Medical Research Council) based funding bodies and prime partners from those countries were common in these initiatives.

### Focus regions and researchers

The NCD-RCS initiatives included in this study covered LMICs in almost in all world regions but with a particular focus on countries in sub-Saharan Africa (7/14), Inter-regional (5/14), South-East Asia and the Pacific region (1/14), Latin America and Caribbean regions (1/14). RCS Collaborations funded by US-based organizations reached most of these regions while those involving Australia-based funding sources were mainly focused on the South-East Asian region. Apart from some of the initiatives that were focused in a single country, most (13/14) were multi-country NCD-RCS initiatives.

The majority (12/14) of the initiatives involved early and mid-career *researchers* (graduates of Masters Degrees, doctoral students and post-doctoral fellows) as their main target groups for capacity strengthening. A few have also engaged health care professionals – e.g. clinicians, nurses and policy makers - working in the field of NCD prevention and control. However, most of the RCS initiatives lacked an explicit multi-sectoral focus and typically, were more focused on the health sector.

### NCDs and risk factors of interest

Most (9/14) NCD-RCS initiatives were *generic* and thus not limited to any specific non-communicable or chronic diseases or risk factor and considered all forms of NCDs. Some (2/14) have areas of *concentration* on specific clusters or combinations of diseases such as cardiovascular and metabolic diseases, heart and lung diseases, diabetes or cancers. Nevertheless, a few (3/14) were also limited to *specific* disease (cancer) or risk factor (tobacco use). Accordingly, the NCD-RCS initiatives were categorized as generic, concentrated, and specific.

### Implementation mechanism

All of the NCD-RCS initiatives involved collaboration among a number of institutional and other partners from other countries and regions to deliver the program. While North-South Collaboration was the most common, there are also more recent examples of North-South-South collaboration and South-South collaborations. As described in Table 1, six out of the 14 initiatives have been completed, while the remaining eight are long-term and ongoing.

### Strategies of RCS

Based on the review of the included NCD-RCS initiatives, we have identified a number of inter-related strategies for capacity strengthening. Many of the initiatives are a hybrid of more than one of these. We summarized these models in Table 2 below. Examples of the models of NCD-RCS initiatives and their brief descriptions are provided in Table 3.

**Table 2:** Description of the identified strategies

Strategies	Brief description
Commissioned Research	Fellows/trainees are provided the responsibility to manage a research or a component of a research project in their own institution or local areas. The capacity builder convenes them at critical stages for the research for training, mentoring and networking. They are expected to deliver outputs of the research. ANPPA is an example of this model.
Exchange & Mentoring	An RCS model where two institutions run an exchange program. Trainees from both institutions visit the other and participate in collaborative research. A team of mentors from both institutions will provide an oversight support to the program. ENCORE has used this model.
Embedded RCS:	Research projects with an embedded RCS program are implemented in LMICs. The integration could be either at specific component of the research or throughout the research process from conception to dissemination. Most of the TOBAC projects have demonstrated this approach. Unlike commissioned research, in this model research activities are not “commissioned” to fellows/trainees.
Collaborative Center	Centers or institutions from different countries become part of a larger collaborative centre which leads the management of a collaborative research project and the associated capacity building efforts within it. By participating in the collaborative research, the individual Centers will build their capacity. The AWI-gen study is a typical example of this model.
Institutional Research Training	Two or more institutions collaborative to design and implement a research training program (short-term, medium-term or long-term) which could be online or face-to-face. The program may include other elements such as institutional capacity building and networking. NCD lifespan projects have used this model.
Funding & Networking	This one usually emerges from funders’ side. Through successful grant application, a researcher or a research team receives funding for the proposed research and joins a research network. The funder may then institute targeted RCS efforts in the network. GACD initiative has applied this model.
Knowledge sharing	An approach that creates a knowledge sharing platform where NCD related knowledge is sourced, stored and shared to researchers, policy-makers and other potential users. By providing better access to up-to-date NCD information, this approach promotes NCD research and enhances NCD research capacity among researchers and evidence use among policy makers. KSN-NCD adopted this model.
Professionals’ network	A network of professionals (individuals) working on NCDs facilitates information exchange and advocacy. It also creates fertile ground for design and implementation of training and research programs within the network. This is a self-led initiative with support from partner institutions. YP-CDN is a typical case of this model.
Potter–Brough model	A systematic capacity strengthening model involving four levels: 1) Systems level: structures, systems and roles; 2) Infrastructure: facilities, resources and staffs; 3) Individual level: knowledge, skills and confidence; and 4) performance level: the availability of resources and tools needed to complete activities. INCAN project applied this model of RCS. The model is relatively more comprehensive but could be resource intensive.
Organizational Capacity Strengthening	This model involves strengthening organization capacity of an institution so that that institution can develop, implement and evaluate RCS projects on its own or in collaboration with others. The model, as implemented by AACR-AORTC, may require an extended support from the organization involved in strengthening capacity.
Collaborative research	Researchers and research institutions work in partnership to design research, look for research funding, implement research and share evidence. This enables researchers to undertake multi-country research projects that could provide internship/fellowship opportunities for early and mid-career researchers. Unlike the collaborative Centre, this one doesn’t involve establishment of a centre.
Multi-Sectoral Research group	NCDs have multiple risk factors and require multi-sectoral action. Forming multi-sectoral research groups, such as the Africa-wide NCD research group, would help address the different perspectives required in NCD research and enhances research capacity of the team in producing better quality research.
Centre of excellence	Creating Centres of excellence in NCD research, networking them to work together and providing them with the responsibility of NCD-RCS are the main principles of this model. This approach seems to ensure sustainability of RCS in LMICs. NHLBI-UnitedHealth Collaborating Centres of Excellence (COE) program used this model.
Consortium	This is an association between two or more research institutions that enable them to involve in common research or pool their resources (including data) for a common research. To cite an example, the CNCD Africa consortium created opportunities for involvement of young researchers in a better NCD research.

**Table 3:** Description by example of the approaches used by the selected NCD-RCS initiatives

SN	NCD-RCS initiatives	Description of models/approaches
1	ANPPA	*Commissioned Research*: Build research capacity on multi-sectorial action for health in Africa and to set up a networked group of researchers in NCDs to monitor and assess the effectiveness and impact of multi-sectorial approaches in the long term in Africa.
2	ENCORE	*Exchange & Mentoring*: Exchanges and mentoring between Australia and India; online training sessions; a supported twice-yearly forum in India/Australia that is attended by the ENCORE Faculty and trainees.
3	TOBAC	*Integrated research*: RCS integrated in to research. A total of 29 individual TOBAC projects ranging from individual training approaches to multi-level and multi-factorial approaches aimed at systemic change by conducting research and training researchers.
4	AWI-Gen	*Collaborative Centre*: Build capacity in Africa for research that leads to an understanding of and response to the interplay between genetic, epigenetic and environmental risk factors for obesity and associated cardio-metabolic disease (CMD) in sub-Saharan Africa.
5	NCD-lifespan	*Institutional Research Training*: Collaborative research training between institutions in the U.S., LMICs and other HICs. The program aims to sustainably strengthen the research capacity of the LMIC institutions, and to train in-country experts through short-term, medium-term and long-term training as well as additional institutional capacity-building efforts.
6	GACD	*Funding and networking*: Fund, develop and facilitate innovative research collaborations between low- and middle-income and high-income countries in the fight against chronic diseases.
7	KSN-NCD	*Knowledge sharing platform*: NCD-info knowledge sharing portal. The Kenya NCD-info portal is designed to be a one-stop portal for all information related to NCDs in Kenya.
8	YP-CDN	*Professionals’ network*: Next-Gen Fellows complete a 1-year fellowship that seeks to build their leadership for improved NCD advocacy, research and policy influence at sub-national, national, regional and global levels.
9	INCAN	*Institutional development*: A multidisciplinary Cancer Control Research Training Institute was developed at the Instituto de Cancerología (INCAN) in Guatemala City. This institute provided a year-long training programme for clinicians that focused on research methods in population health and sociocultural anthropology.
10	AACR-AORTC	*Research alliance*: Africa Cancer research Alliance running workshops designed to facilitate improved expertise, resources and infrastructure that can lead to impactful investigator-initiated cancer research in Africa.
11	APCDR	*Collaborative Research*: APCDR is an international research partnership that assesses the burden and causes of non-communicable diseases such as diabetes and heart disease in sub-Saharan Africa. It allows researchers from the partnership to develop a sustainable platform to share resources and skills.
12	Africa wide NCD Research Group	*Multi-sectoral Research group*: The research group will facilitate NCD research in a comprehensive, multi-sectoral and coordinated approach by tapping into a region-wide pool of multi-disciplinary teams.
13	Training and Capacity Building in LMIC for Research in Heart and Lung Disease	*Centres of Excellence*: A network of 11 collaborating COEs based in institutions from 10 LMIC who partnered with research institutions from high-income countries. Strategies included academic degree programs, non-degree credential programs, mentoring, conferences, workshops and other educational approaches. COEs to take over the RCS initiative.
14	CNCD-Africa	*Research Consortium:* CNCD-Africa aims to comprehensively address specific and common objectives while building capacity in the region to prevent and control NCDs. CNCD focuses on four key areas: convening; knowledge generation and sharing; advocacy; and networking.

### Capacity areas addressed

While most of these approaches or strategies focused on building individual and team level NCD research capacity, less than half of the initiatives have also claimed institutional-level capacity building. A limited number (4/14) have included efforts to strengthen capacity at the research network level, as well. However, only one initiative had addressed research capacity at all levels.

Strengthening capacity in conducting research was the focus of the reviewed NCD-RCS initiatives. Engagement with potential users of research to promote use of evidence has received less attention. Research capacity in increasing the breadth (involving multiple countries) and depth of understanding of NCD epidemic was underpinning most of the NCD-RCS approaches.

Almost all the NCD-RCS focused on enhancing research or research engagement capacity with little direct action on NCD management skills. The research skill areas addressed are generic. But for some of the initiatives it was on specific NCD research as the funding streams were specific to a disease or a risk factor.

## Discussion

### Summary of the findings

This study has identified that many new and hybrid models of NCD-RCS are emerging in low and middle-income countries in recent years, especially in sub-Saharan Africa and Asian countries. With the increasing priority being accorded to NCD prevention and control in these settings in terms of policy and resource allocations [46], this is a positive development that has the potential to contribute to a narrowing of the evidence-implementation gap, thereby contributing to the realization of the global NCD targets.

### Interpretation of the findings

Multi-faceted strategies of NCD training and capacity strengthening are becoming more common within countries and there is less reliance on long periods of overseas training. The most common strategies included face-to-face sessions, online training and learning by undertaking research. Internet and new technologies are being utilized more overtly in program delivery over time. Recent initiatives are considering the use of internet and new technologies; the collaboration between institutions in LMICs and high-income countries; and involvement of the diaspora in reducing ‘brain drain’ which was a problem in traditional overseas scholarships and fellowships. Some initiatives also provided some financial support for undertaking research.

Although we have provided a brief description of the common approaches used in NCD-RCS, it was not possible to identify which model was or could be more/less effective than the other in terms of improving research performance and quality of research outputs. This was due to the fact that evidence on outcomes of these initiatives was not readily available. Evaluation of outcomes of RCS programs is complex and even if evaluations are conducted they can only demonstrate short-term and immediate outcomes [8, 47]. In this regard, future NCD-RCS initiatives need to integrate evaluation studies that can demonstrate long-term outcomes of such initiatives.

On the other hand, there is little evidence to ascertain the continuity and sustainability of the results of these initiatives. Tracing the outputs of the trainees after completion of the program was not part of most of the initiatives and evidence on continuing engagement of the trainees in research is lacking. One plausible reason for this could be the nature of funding of the initiatives. As shown in this study, most of the NCD-RCS initiatives were funded by external donors. This can affect the sustainability of the initiatives as local systems may not continue implementing the initiatives after the funding is withdrawn [48]. Moreover, country ownership of the initiatives can only be ensured with full participation of the local systems in the design, implementation and evaluation of these initiatives [49].

### Implications of the findings

The models/approaches used by NCD-RCS initiatives share many similarities with that of RCS in other health areas like communicable diseases. As the generic research skills required for NCDs and chronic communicable diseases overlap, the NCD-RCS initiatives can learn from evidence on RCS in chronic infectious diseases such as HIV/AIDS and Tuberculosis [50]. Not only the lesson, but also researchers trained in chronic infectious disease research, through a tailored research mentoring program, could be motivated to engage in NCD research in LMICs. This would be of significant importance in research on co-morbidities between communicable and NCDs.

Most of the NCD-RCS initiatives have focused on strengthening individual level capacity. However, very little has been done to improve institutional and system level capacity. Without a meaningful change in institutional research infrastructure and system level research culture, it will be challenging to improve performance of researchers, quality of research outputs and more importantly the utilization of research findings for policy and action [51]. Therefore, a more comprehensive and integrated approach to NCD-RCS is needed in LMICs.

We observed a modest increase in the number of NCD-RCS initiatives in LMICs across years [21]. However, whether NCD research training programmes are closely linked with the national NCD prevention and control agenda in LMICs is not well examined and the establishing locally relevant and harmonized NCD research capacity strengthening agenda is the main priority in the foreseeable future. Given the significant gap between research evidence and public health priorities LMICs, formulating NCD RCS agenda would also help bridge this gap between NCD research and implementation of NCD programs [52].

### Limitations of the study

This study used multiple sources of information and applied a deeper analysis of information to identify and describe NCD-RCS models. However, there were some limitations associated with this review. Firstly, this review largely used publicly available evidence as its main source of information. Consequently, our resulting models may not include all the available ones. Secondly, in this review, we used a mix of peer-reviewed literature and grey literature to identify and describe the approaches and we did not undertake any quality assessment of the evidence. Thirdly, the initiatives included in this study vary from single projects to networks and bigger funding schemes. There are several smaller projects within the bigger funding schemes that have various focus areas. These were represented by the overall model/approach at scheme level. Fourthly, with the inclusion criteria of programs at least one year in length, it is likely to have missed many but important initiatives with shorter duration. Furthermore, since a lot of the research capacity building activities are not published in peer-reviewed journals, it is likely to have missed several important ones. Finally, there was a lack of information in the data sources of the selected NCD-RCS initiatives as the study highly relied on secondary information.

## Conclusions and recommendations

In this study, we identified and described several different approaches of NCD-RCS as applied by the initiatives included in the review. Diverse and hybridized approaches of NCD-RCS initiatives have been implemented in many LMICs. However, information on program design, implementation and evaluation of these initiatives is inadequate. Consequently, the relative effectiveness and cost-effectiveness of these initiatives remain largely unknown. Moreover, given the external funding of these initiatives, the sustainability of the NCD-RCS initiatives at local level remains a critical concern. Proper documentation and evaluation of NCD-RCS initiatives would enhance the outcomes and implementation of NCD-RCS initiatives.

## Data Availability

Not applicable.

## Appendix 1

**Table 4:** Sample Search strategy (Embase)

#	Searches
1	exp Non-communicable disease /
2	limit 1 to (english language and yr = “2000–2017”)
3	exp research/
4	limit 3 to (english language and yr = “2000–2017”)
5	(capacity strengthening OR capacity building OR training).mp. [mp = title, abstract, heading word, drug trade name, original title, device manufacturer, drug manufacturer, device trade name, keyword, floating subheading word, candidate term word]
6	limit 5 to (english language and yr = “2000–2017”)
7	(low and middle-income countr*).mp. [mp = title, abstract, heading word, drug trade name, original title, device manufacturer, drug manufacturer, device trade name, keyword, floating subheading word, candidate term word]
8	limit 7 to (english language and yr = “2000–2017”)
9	developing countr*.mp.
10	limit 9 to (english language and yr = “2000–2017”)
11	8 or 10
12	2 and 4 and 6 and 11

## Appendix 3

**Table 5:** Data Extraction Template

SN	Key parameters	Information extracted
1	Full title of the initiative	
2	List of implementers and partners & their roles	
3	Region and countries of implementation	
4	Implementation Period Years) and duration of initiative	
5	Targeted researchers for capacity strengthening	
6	Focus area in terms of disease (if any)	
7	Skill areas considered for capacity strengthening	
8	Capacity strengthening strategies applied and implementation mechanism (abstracted)	
9	Funding support/list of donors supported the initiative	
10	Data sources	

## Abbreviations

AACR-AORTC: American Association for Cancer Research and African Organisation for Research and Training in Cancer (AACR - AORTIC)
AIIMS: All India Institute of Medical Science
ANPPA: Analysis of Non-communicable Diseases Prevention Policies in Africa
APCDR: African Partnership for Chronic Disease Research
APHRC: African Population and Health Research Center
ASCEND: ASian Collaboration for Excellence in Non-Communicable Disease
CCCC: Centre for the Control of Chronic Conditions
CDC: Center for Disease Control
CNCD: Consortium for Non-Communicable Disease
ENCORE: Excellence in NonCOmmunicable disease Research
FIC: Fogarty International Center
GACD: The Global Alliance for Chronic Diseases
HHS: Health and Human Services
IDRC: International Development Research Centre
INDEPTH: International Network for the Demographic Evaluation of Populations and their Health
IUPHE: International Union for Health Promotion and Education
KSN-NCD: Knowledge Sharing Network on Non-communicable Diseases
MRC: Medical Research Council, UK
NHLBI: National Heart, Lung, And Blood Institute
NIDA: National Institute on Drug Abuse
NIH: National institute of Health
PHFI: Public Health Foundation India
RLTP: Research Leadership Training Program
RTI: Research Triangle Initiative
SCTIMST: Sree Chitra Tirunal Institute for Medical Sciences and Technology
TOBAC: International Tobacco and Health Research and Capacity Building Program
YP-CDN: Young Professional Chronic Disease Network
